# Supplementary material for: A graph-based genome and pan-genome variation of the model plant Setaria
Source: Nat Genet. 2023 Jun 8;55(7):1232–42. doi: 10.1038/s41588-023-01423-w (PMC10335933; doi:10.1038/s41588-023-01423-w)
Supplement: Supplementary file 1 — Supplementary Notes 1–5 and Supplementary Figs. 1–17. [file 41588_2023_1423_MOESM1_ESM.pdf]

nature genetics

Article

https://doi.org/10.1038/s41588-023-01423-w

A graph-based genome and pan-genome variation of the model plant *Setaria*

In the format provided by the authors and unedited

Supplementary Note 1: Population features of *Setaria*. 2
Supplementary Note 2. The representative of 110 Setaria accessions for pan-genome study. 3
Supplementary Note 3: Examples of structure variations in gene regions. 4
Supplementary Note 4: PAVs associated with gene expression. 5
Supplementary Note 5: QTL mapping for seed shattering of foxtail millet 6
Supplementary Figures 7
Supplementary References 25

Supplementary Note 1: Population features of *Setaria.*

Within the domesticated species, both TREEMIX[1] and Admixtools[2] show that the first evolutionary split is between C3 and subgroups C1/C2, with the latter two diverging later (**Supplementary Fig. 2**). Using these two approaches as well as f4 statistics[2] (**Supplementary Table 3**), we found evidence for gene flow between some cultivated and wild subpopulations, although we cannot as yet ascertain its adaptive impact on domesticated foxtail millet. Nevertheless, we are able to use Graph-aware Retrieval of Selective Sweeps (GROSS)[3] to scan for genomic regions under selection along the branches leading to each of the cultivated subpopulations. We identified 52 selected genomic regions along the terminal branches leading to the different cultivated subpopulations – 27 for C1, 14 for C2 and 11 for C3 - which may be associated with local adaptation in foxtail millet (**Supplementary Fig. 2 and Supplementary Table 4**).

To better understand the characteristics of subpopulations within *Setaria*, we conducted analysis of nucleotide diversity, heterozygosity and F_{ST} for these subgroups. Nucleotide diversity and heterozygosity analysis showed that C2 has the lowest diversity (1.73×10^{-3}) and average heterozygosity (0.048) (**Supplementary Fig. 3d**). Pairwise F_{ST} analysis indicate that C1 and C2 is closer to each other compared to other subgroups (**Supplementary Fig. 3e**).

Supplementary Note 2. The representative of 110 Setaria accessions for pan-genome study.

To capture the full spectrum of genetic diversity of *Setaria*. i) We selected accessions based on phylogenetic relationships and geographic distribution; ii) we also selected accessions which have contributed significantly to foxtail millet breeding and/or research, such as breeding backbone parent lines (Liushiri and Ai88), accessions with high eating and cooking quality (Jingu21 and Huangjinmiao), high drought tolerance (Zhonggu 2), wide climate adaptation (Yugu 18), and easy transformation (Ci846). According to population structure analysis, 22, 25, 5, 28, 1, and 1 accessions belong respectively to the C1, C2, C3, W1, W3 and W4 subgroups, and 28 accessions are admixed (**Supplementary Table 5**). Finally, these accessions covered over 85% of SNP variation across the 1,844 *Setaria* accessions.

Supplementary Note 3: Examples of structure variations in gene regions.

Structure variation can significantly impact on gene function. We found a set of SVs that localize within promoters or gene bodies of functionally important loci. For example, a 124 bp deletion impacted the promoter and first exon of *Seita.1G213000*, a homolog of the cytochrome P450 gene *D11* involved in brassinosteroid biosynthesis that in rice regulates grain shape, grain weight and plant height[4]. We also observed a 1.4 kb deletion within the gene body of *Seita.2G276500*, a homolog of rice disease resistance-related *OsSGT1* gene[5]; a 627 bp deletion present in the first intron of *Seita.7G171000*, a homolog of rice plant height, tillering and root development-related gene *OsGA2ox6*[6]; and a 2.02 kb deletion causing loss of *Seita.8G046900* and *Seita.8G047000*, homologs of *OsMYB2P-1*, which are involved in regulation of rice phosphate-starvation responses and root architecture[7]. Moreover, a 360-kb inversion identified in Ci846 contained the foxtail millet ortholog of *FLO*, a gene controlling grain size and starch quality in rice[8] (**Supplementary Fig. 5e**).

Supplementary Note 4: PAVs associated with gene expression.

PAVs are thought to be important drivers in regulating gene expression[9]. Using transcriptome sequencing data from ten tissues of the "Yugu1" accession, we explored expression patterns of 7,191 genes carrying common PAVs within 2-kb upstream of their coding sequence and present in >10% of accessions. We find that expression levels of PAV-associated genes were generally lower than those without PAVs (two tailed student's *t*-test, $p = 2.2 \times 10^{-16}$) (**Supplementary Fig. 6a-b**). Gene ontology (GO) enrichment analysis showed that these PAV-associated genes were enriched in regulation of developmental processes, response to abiotic stress, and auxin homeostasis (**Supplementary Fig. 6c**).

Interestingly, the density of differentially expressed genes (DEGs) show significantly positive correlation with PAV density between both wild and cultivated foxtail millets (A10 versus Yugu1, *R* ranging from 0.42 to 0.64) and between landrace and modern cultivated accessions (Ci846 versus Yugu1, *R* ranging from 0.41 to 0.49) (**Supplementary Figs. 6d-e**).

Supplementary Note 5: QTL mapping for seed shattering of foxtail millet.

To identify seed shattering loci, we constructed a recombinant inbred line (RIL) population using a cultivar (Yugu1, non-seed shattering) and a wild accession (Q24, shattering) as parents (**Supplementary Fig. 11a**). Both bulked segregant analysis sequencing (BSA-seq) and QTL analysis identified three major QTLs (*qSH5.1*, *qSH5.2*, and *qSH9.1*) controlling seed shattering in *Setaria* (**Supplementary Figs. 11b-c**).

For *qSH9.1*, we fine-mapped and confirmed QTL function using two independent RILs, which displayed different seed shattering phenotypes and different genotypes at *qSH9.1*, but share the same genotype at *qSH5.1*, *qSH5.2*, and have 84% genome-wide sequence similarity; these were selected to construct near-isogenic lines (NILs) by backcrossing for three generations and self-pollinating for two rounds. Then we narrowed the qSH9.1 into an 87.3kb region between markers M2 and M3, which contained *Seita.9G154300* (*sh1),* a homologue of the rice shattering gene *OsSh1*.

Supplementary Figures

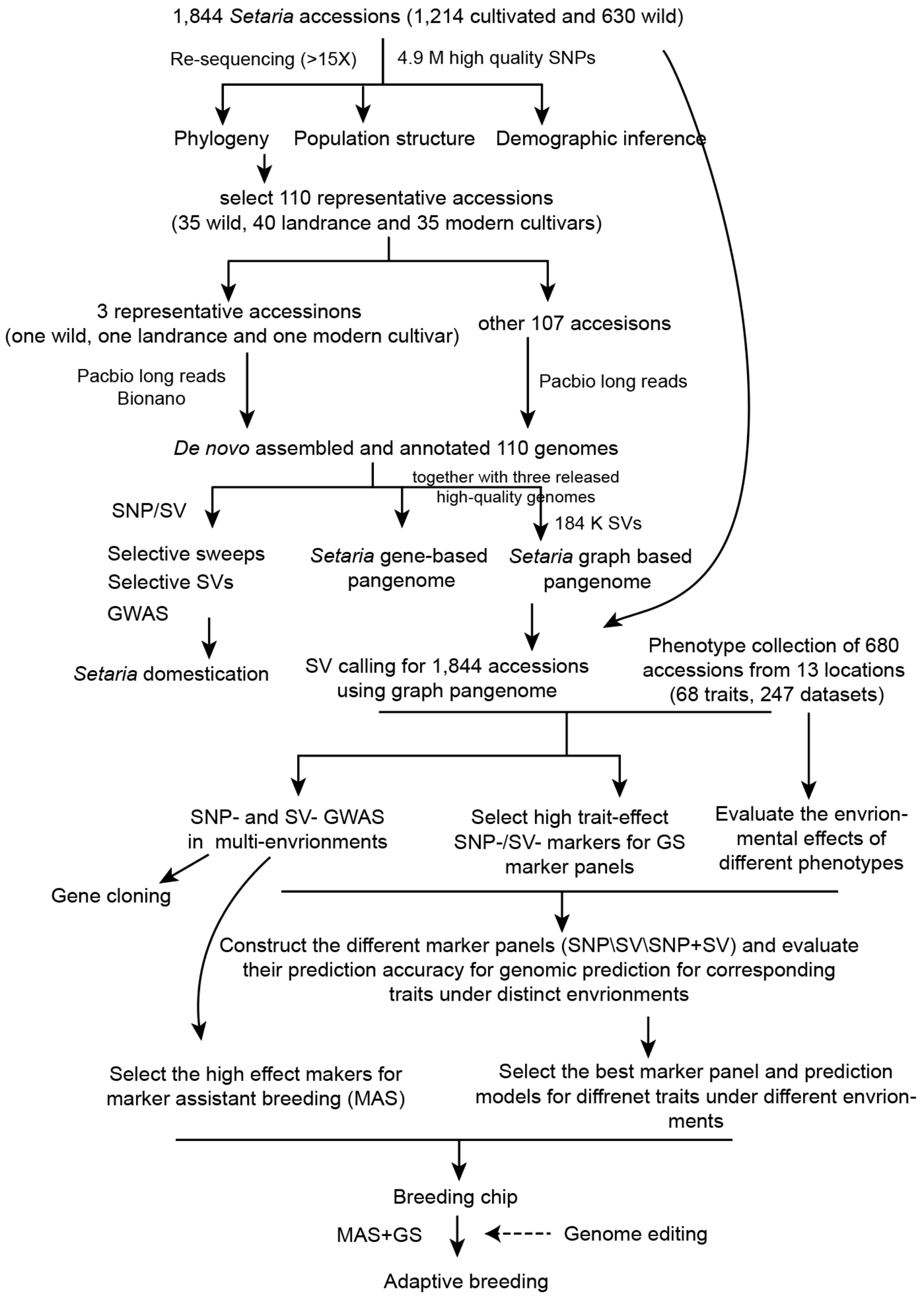

Supplementary Fig. 1. The schematic of experimental design of this study.

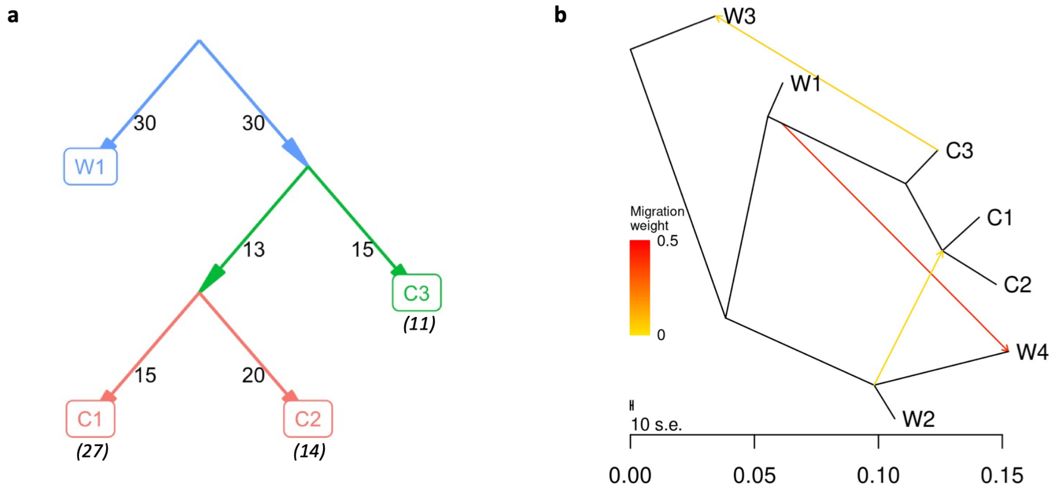

Supplementary Fig. 2. Population relationships among wild and domesticated subpopulations and evidence for local adaptation. a. The topology and relationships between the domesticated subpopulations C1, C2 and C3 and the closest wild population W1. The numbers next to the arrows are the scaled drift parameter based on *f2* statistics, and those in bracket next to the subpopulation names represent the number of selected genome regions for the three cultivated subpopulations. The coordinates of the regions putatively under selection are provided in **Supplementary Table 4**. **b.** Population admixture graphs including all seven subpopulations were inferred using TreeMix with W3 as outgroup. The yellow and red arrows represent possible gene flow events between wild and cultivated subpopulations.

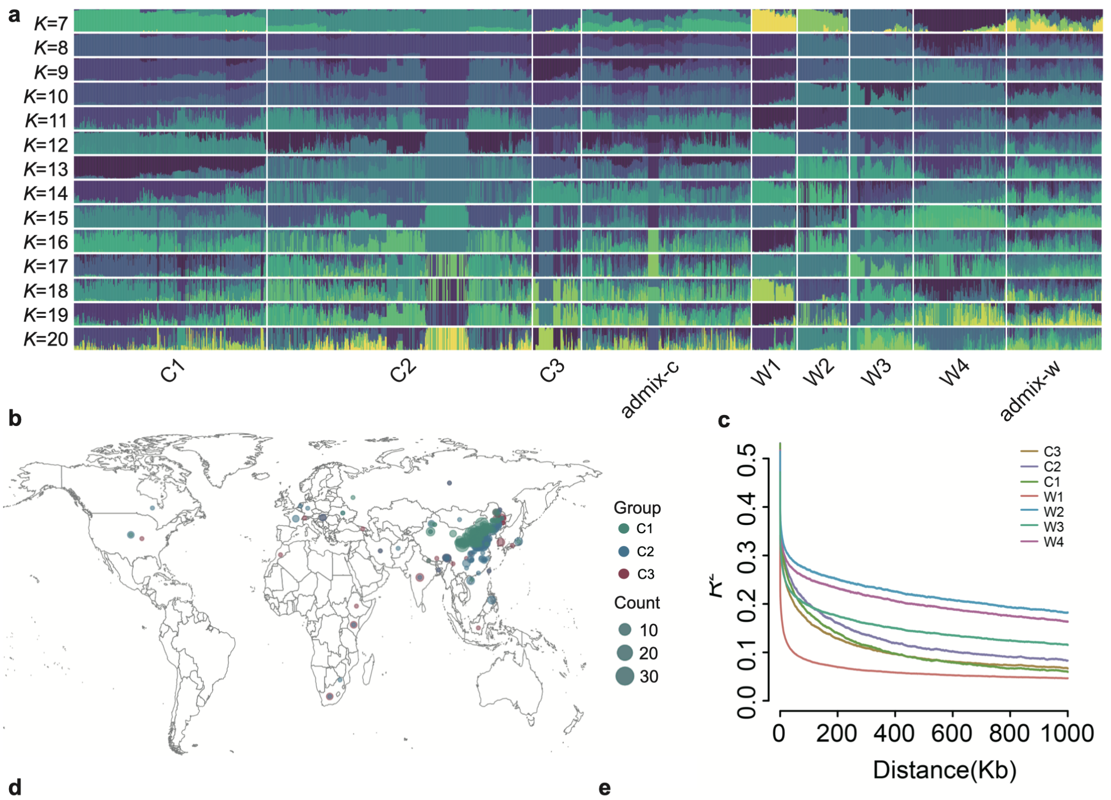

d

Pop	Nucleotide diversity (10^{-3})	Observed hetrozygosity
C1	1.91	0.070
C2	1.73	0.048
C3	2.09	0.065
W1	2.94	0.049
W2	1.93	0.049
W3	2.41	0.051
W4	2.07	0.055

e

Pop	C1	C2	C3	W1	W2	W3
C1						
C2	0.094					
C3	0.133	0.158				
W1	0.293	0.324	0.205			
W2	0.377	0.398	0.332	0.178		
W3	0.391	0.411	0.327	0.224	0.303	
W4	0.375	0.392	0.350	0.197	0.137	0.325

Supplementary Fig. 3. Population characteristics of *Setaria*. a, Population structure of *Setaria* from *K*=7 to *K*=20; **b**, Geographic distribution of three subgroups of foxtail millet. The map was created using the map_data() function in the R package ggplot2; **c**, Linkage disequilibrium decay of seven subgroups of *Setaria*; **d**, Nucleotide diversity (*Pi* value) and average observed heterozygosity for each subgroup; **e**, F_{ST} for pairwise subgroups.

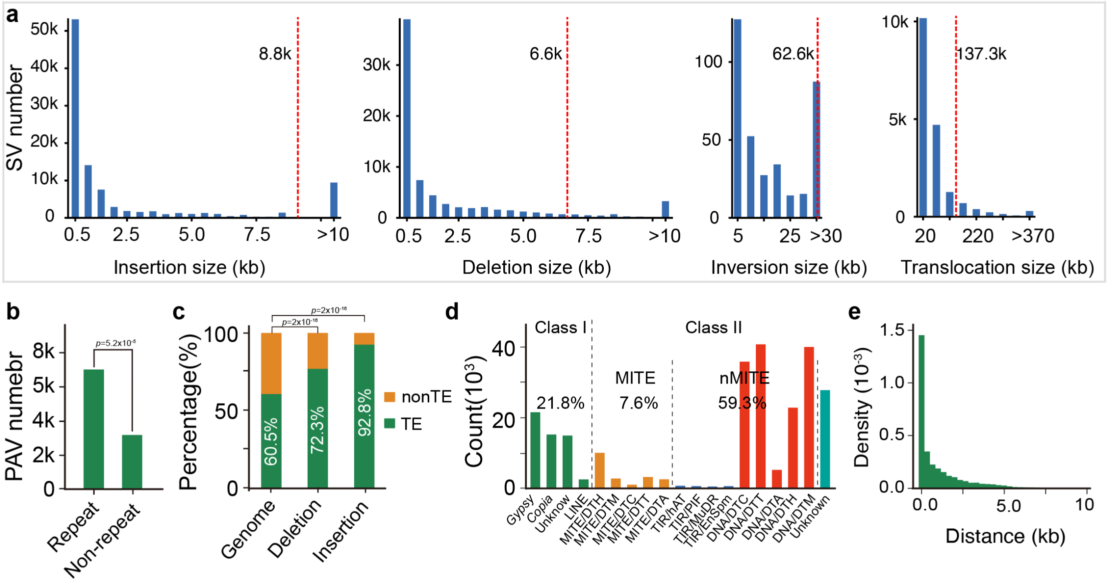

Supplementary Fig. 4 Number, size and distribution of different types of structural variants (SVs) and transposable elements (TEs). a, Size distribution of different types of SVs; the dashed red lines represent the length of the SV reaching 90% of the whole dataset. **b,** The number of PAVs containing repeats is significantly higher than those without repeats (Significance are computed in two-sided Student's *t*-test). **c,** Percentage of TE and nonTE regions of reference genome and PAVs (Significance are computed in two-sided Fisher's exact test). **d,** Proportions of TE annotated PAVs. **e,** Distribution of PAV density against the distance between PAV breakends and junction sites of the PAV-overlapped TE.

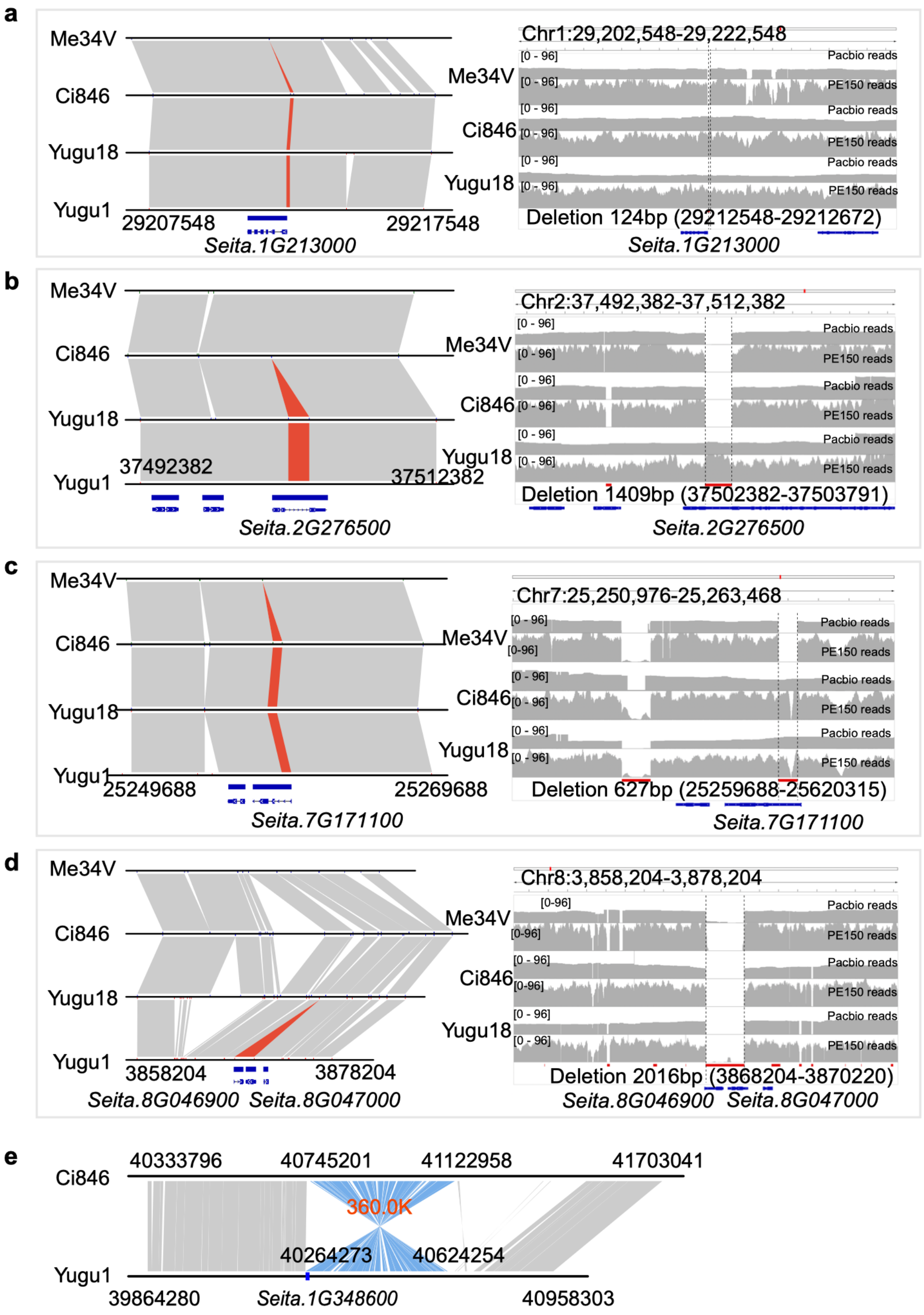

Supplementary Fig. 5 Structural variants detected in five case genes. a, A 124-bp deletion localized in the first exon and promoter region of *Seita.1G213000* (left), which was verified by long-read and PE150 short read mapping (right). **b,** A 1409 bp deletion in gene body of *Seita.2G276500* (left) verified by long-read and PE150 short read mapping (right). **c,** A 627 bp deletion in the first intron of *Seita.7G171000* (left), which was verified by long-read and PE150 short read mapping (right). **d,** A 2016 bp large deletion results in the loss of *Seita8G046900* and *Seita.8G047000* (left), which was verified by long-read and PE150 short read mapping (right). **e,** A 360-kb inversion occurred at *Seita.1G34860*.

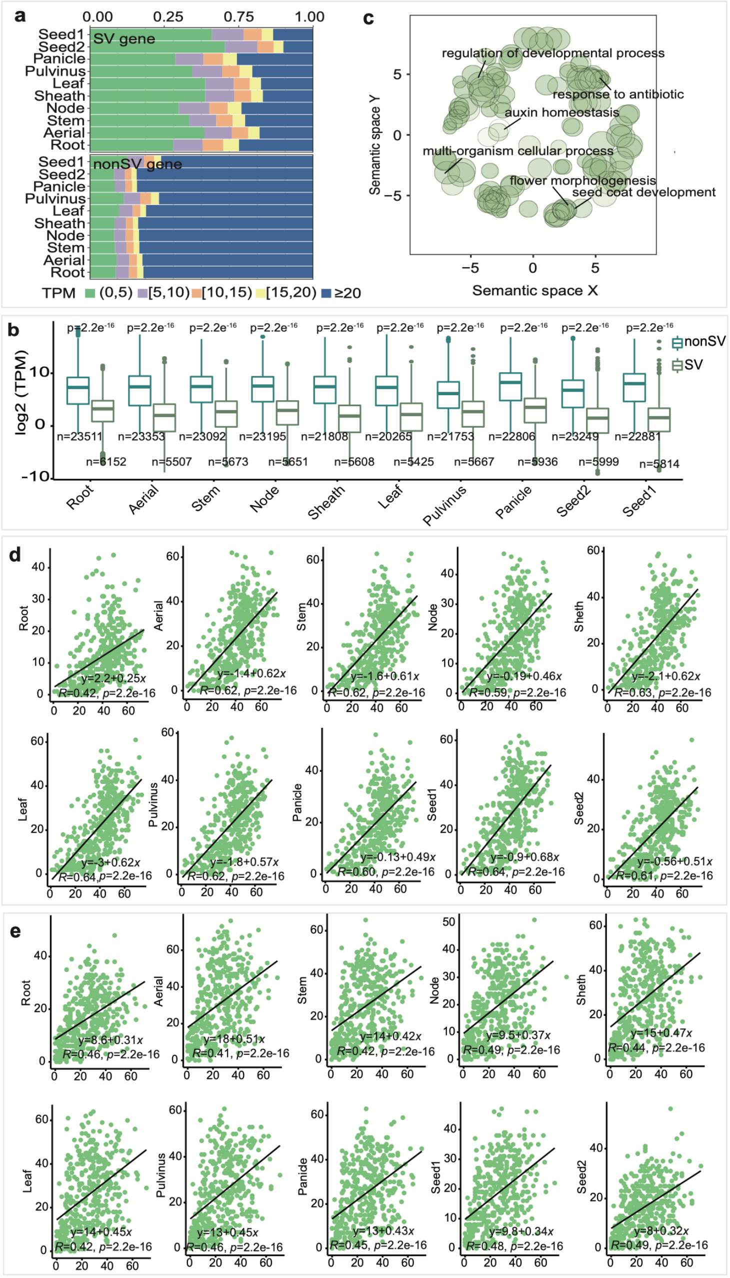

Supplementary Fig. 6 SVs impact gene expression in different tissues. a, Proportion comparison between SV-genes and nonSV-genes expression across 10 tissues from Yugu1. Seed1 and seed2 indicate the matured seeds and filling stage seeds, respectively. **b**, Expression level of SV associated and non-SV gene in different tissues (p-value = 2.2×10^{-16}, two-tailed Student's t-test for TPM). **c**, Enriched GO items of SV-genes. **d**, Correlation between DEG (A10-vs-Yugu1) and PAV density of different tissues across 1-Mb interval in genome. **e**, Correlation between DEG (Ci846-vs-Yugu1) and PAV density of different tissues across 1Mb interval in genome. Significance in d and e are tested with two-sided Student's t-tests.

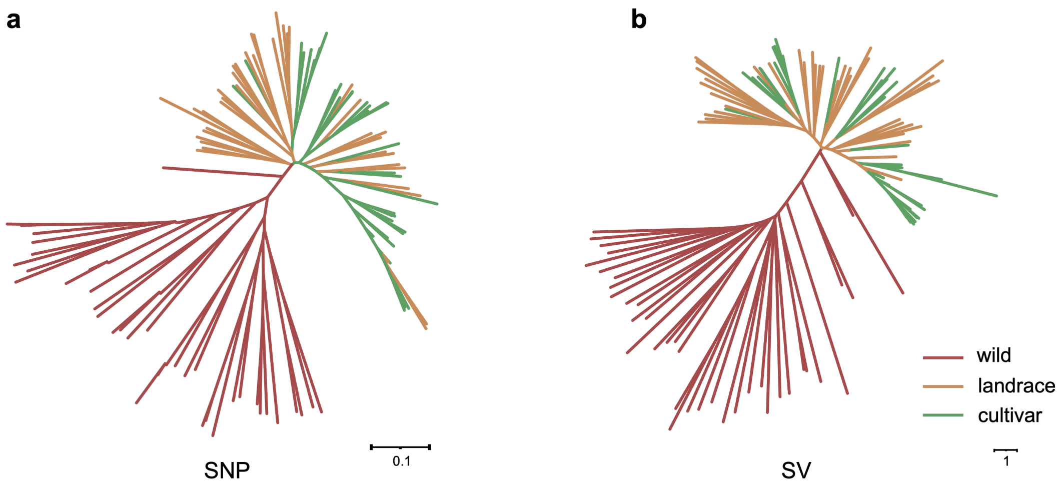

Supplementary Fig. 7 Phylogenetic tree of the 112 accessions using polymorphism information of SNP and SV. a, SNP-based phylogenetic tree. **b,** SV-based phylogenetic tree.

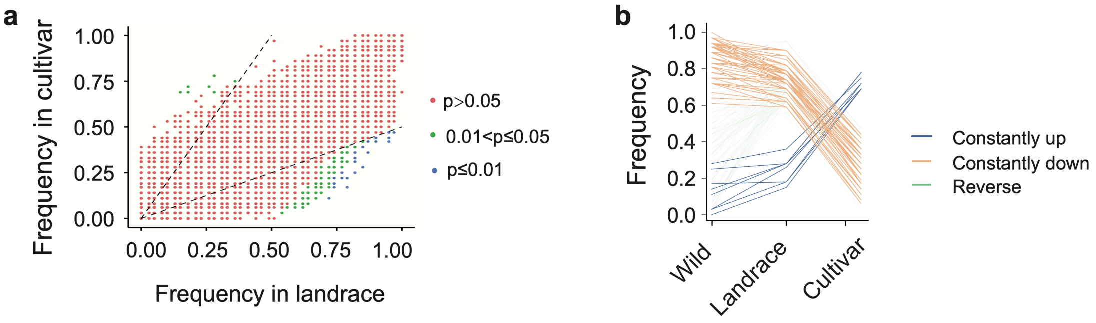

Supplementary Fig. 8 PAV selection preference during improvement of foxtail millet. a, Scatter plots show SV frequencies in landrace and modern cultivars. *P*-value are computed using two-sided Fisher's exact test. **b**, The frequency pattern of improvement related PAVs (impPAVs). Lines with colors of orange and blue indicate fav-PAVs during improvement.

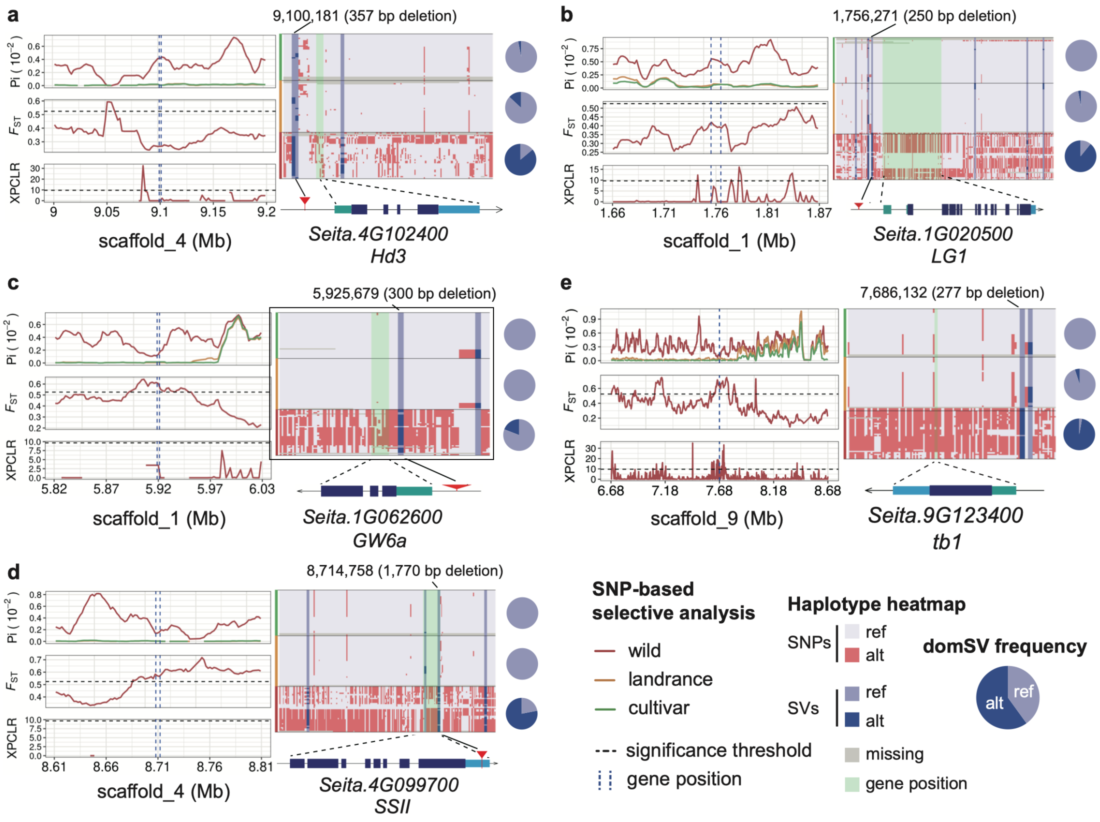

Supplementary Fig. 9 Local SNP-based selective sweep analysis and SV-based selective analysis for representative foxtail millet domestication genes. a-e, SNP based selective analysis around domestication genes using nucleotide diversity (pi), F_{ST} and XP-CLR (left); heatmaps of haplotypes (middle) and domSV frequency (right) changed during the corresponding domestication process (wild to landrace and cultivars). Genes are annotated by homologs of rice (*Hd3*, *LG1*, *GW6a* and *SSII*), and maize (*tb1*). The black horizontal dashed lines at F_{ST} and XP-CLR analysis represent the significance thresholds. *Hd3* and *LG1* are only detected by SV-based selective analysis, while *GW6a*, *tb1* and *SSII* were detected by both SNP- and SV- based selective analysis.

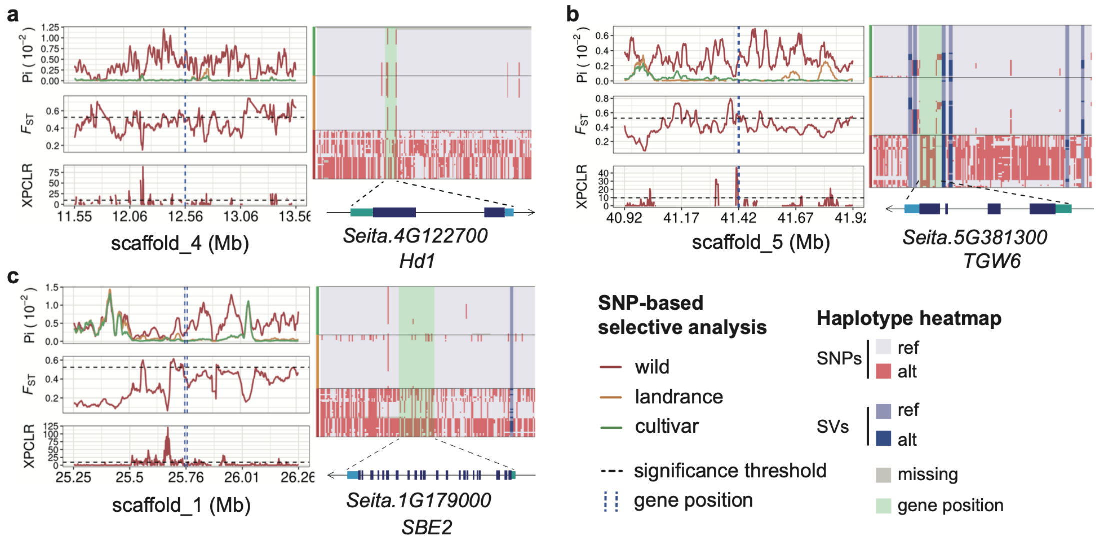

Supplementary Fig. 10 Local SNP-based selective sweep analysis and haplotype analysis for representative foxtail millet domestication genes. a-c, SNP based selective analysis around domestication genes using nucleotide diversity (*pi*), F_{ST} and XP-CLR (left); heatmaps of haplotypes (right) changed during the corresponding domestication process (wild to landrace and cultivars). Genes are annotated by homologs of rice. The black horizontal dashed lines at F_{ST} and XP-CLR analysis represent the significance thresholds. *Hd1* and *TGW6* are detected by *Pi* and F_{ST} analysis, and *SBE2* is detected by *Pi* analysis.

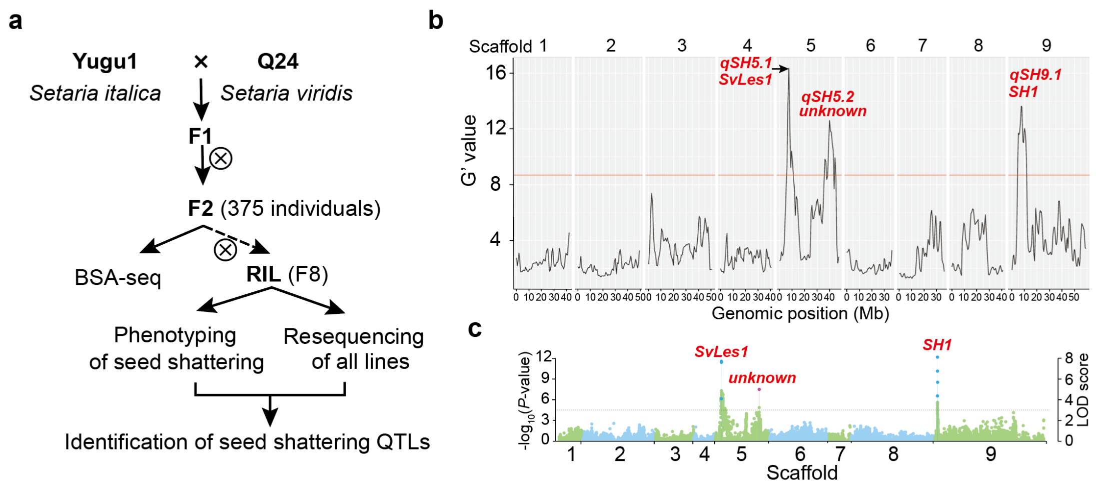

Supplementary Fig. 11 Identification of seed shattering QTLs in *Setaria*. a, RIL population containing 375 individual lines were derived from *S. italica* (non-shattering) accession Yugu1 and *S. viridis* (seed shattering) accession Q24. **b,** In F_2 population, we selected 30 extremely seed shattering individuals and 30 non-shattering individuals to constructed 2 independent DNA pools, and send for WGS of 30x coverage of the genome, respectively. BSA-seq analysis was performed using QTLseqr (https://github.com/bmansfeld/QTLseqR). **c,** QTL analysis using RILs (The significance is obtained from a likelihood ratio test, with the threshold of LOD=3.0). DNA of each 375 RILs in F_8 generation was extracted and send for resequencing (5x coverage for each). Phenotyping of seed shattering were investigated in five different environments. QTL analysis were performed by both rqtl (https://rqtl.org/) and mrMLM (https://cran.r-project.org/web/packages/mrMLM/index.html) methods.

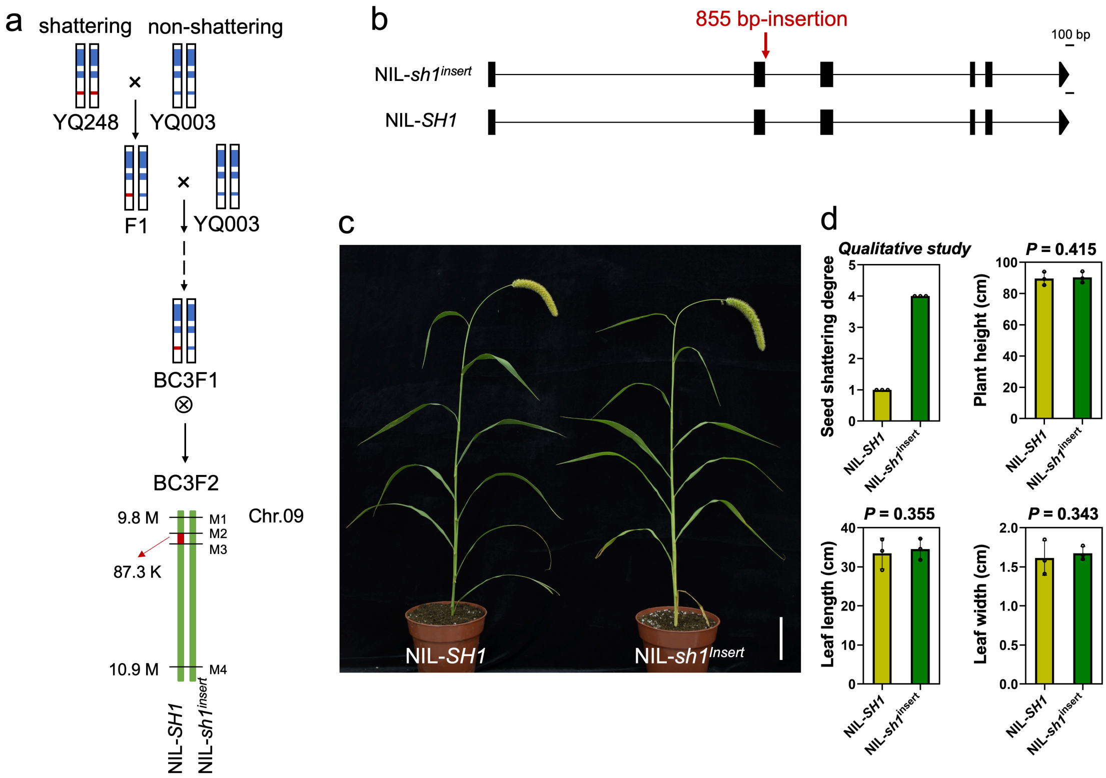

Supplementary Fig. 12. Identification and characterization of *SH1* locus using NILs. a, The schematic of the construction of NILs. YQ003 (non-shattering parent) and YQ248 (seed shattering parent) were selected from Yugu1×Q24 RIL population. The two lines share high similarity in genome backgrounds including the same non-shattering genotype in *qSH5.1* and *qSH5.2* locus. Two NILs were selected in BC_3F_2 population. M1 to M4 are four molecular markers used for fine-mapping. **b,** Gene structure of *sh1* in two NILs. A 855 bp insertion was identified at the end of second exon of *sh1*. **c,** Plant architecture of NIL-*SH1* and NIL-*sh1*^{insert}, bar = 15 cm. **d,** Bar plots of major agronomic trait for NIL-*SH1* and NIL-*sh1*^{insert}. Three biological replications were used for measurements. Non-shattering scales were divided into 5 levels. Level 1 represent seed shattering trait in the most easily shattering accessions such as *S. viridis* Q24, and Level 5 represent the trait in the most non-shattering accessions such as *S. italica* Yugu1. Data are presented as mean±SD in **d**; *P*-values are computed in two-tailed Student's *t*-test.

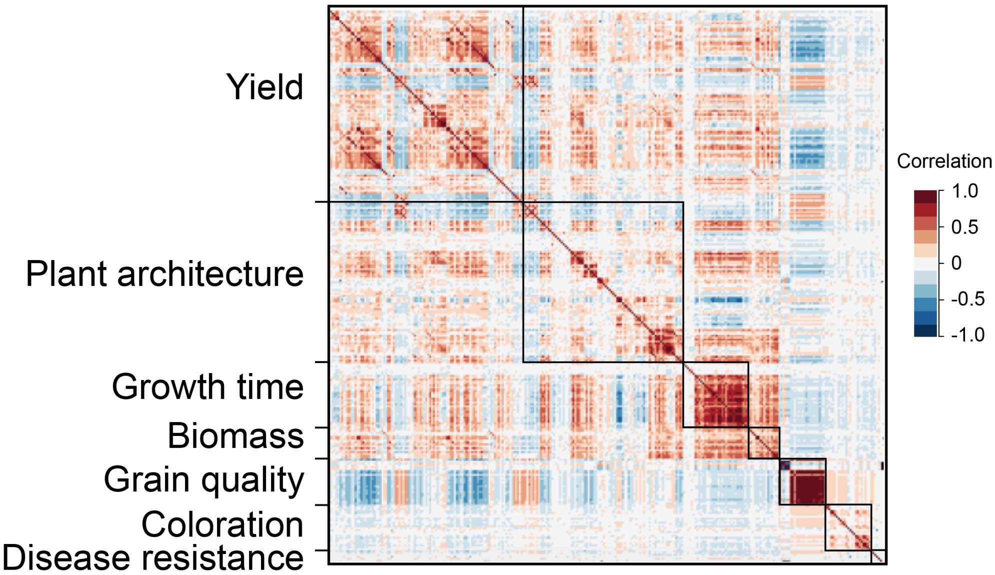

Supplementary Fig. 13 Phenotypic correlation among all 226 sets of phenotypes.

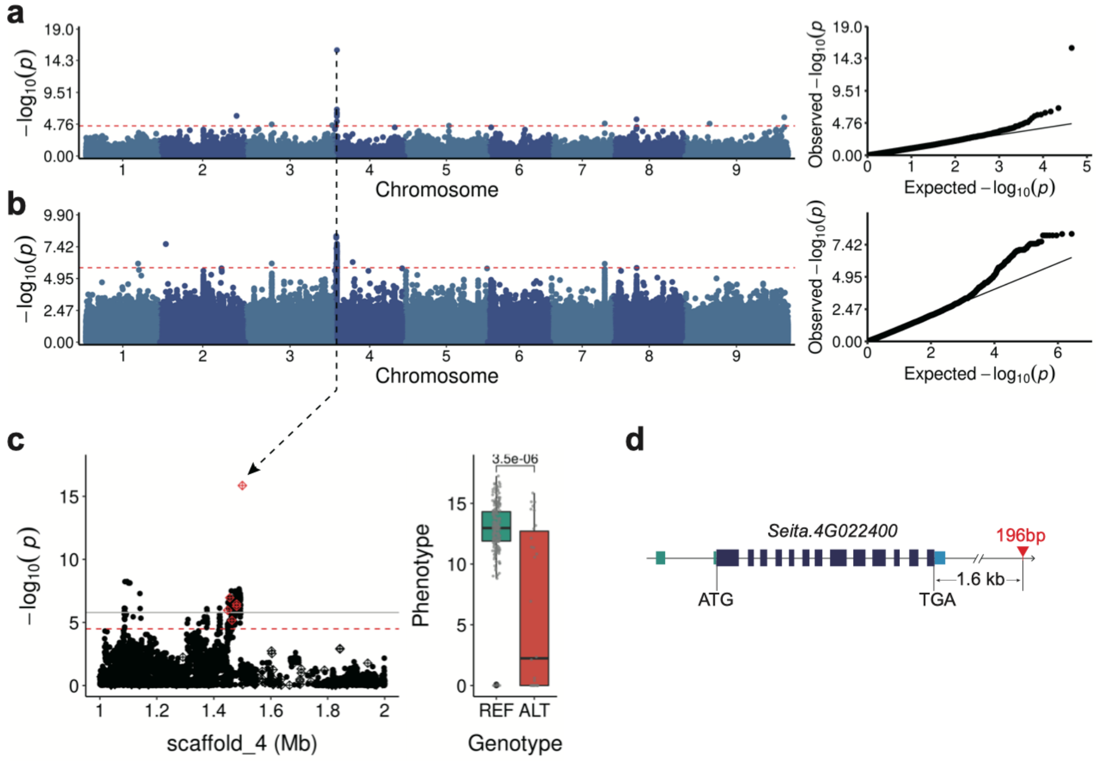

Supplementary Fig. 14 Genome-wide association study of apparent amylose content (AAC) of grain of foxtail millet. a, Manhattan plot and QQ-plot of GWAS results of AAC using SV markers. **b,** Manhattan plot and QQ-plot of GWAS results of AAC using SNP markers. **c,** Scatter plot of the peak-SV in chromosome 4 and boxplot of AAC in different accessions carrying different alleles. Red diamonds and black points indicate PAVs significant associated with AAC and SNPs association-level ($-\log_{10}(p)$) with AAC, respectively. The horizontal line indicates the Bonferroni-corrected genome-wide significance threshold ($\alpha = 1$, and $\alpha = 0.05$). **d,** The peak-SV and gene structure of *Waxy* gene (*Seita.4G022400*). Numbers of samples for REF and ALT in boxplots of **c** are 211 and 30, respectively. The horizontal line indicates the Bonferroni-corrected genome-wide significance threshold ($\alpha = 0.05$) in a and b.

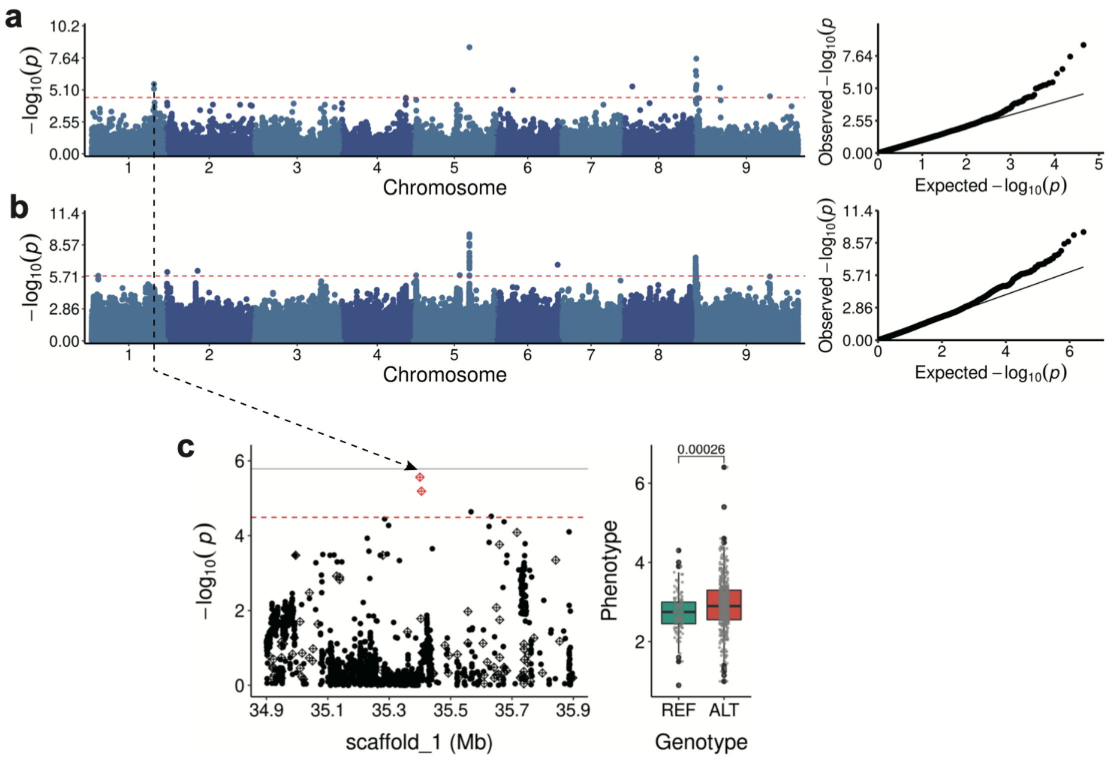

Supplementary Fig. 15 Genome-wide association study of thousand grain weight (TGW). **a**, Manhattan plot and QQ-plot of GWAS result of TGW using SV markers. **b,** Manhattan plot and QQ-plot of GWAS result of TGW using SNP markers. **c,** Scatter plot of the peak-SV in chromosome 1 and boxplot of TGW in different accessions carrying different alleles. Red diamonds and black points indicate PAVs significant associated with TGW and SNPs association-level ($-\log_{10}(p)$) with TGW, respectively. The horizontal line indicates the Bonferroni-corrected genome-wide significance threshold ($\alpha = 1$, and $\alpha = 0.05$). Numbers of samples for REF and ALT in boxplots of **c** are 132 and 529, respectively. In boxplots, the 25% and 75% quartiles are shown as lower and upper edges of boxes, respectively, and central lines denote the median. The whiskers extend to 1.5 times the inter-quartile range. Data beyond the end of the whiskers are displayed as black dots. *P*-values were computed from two-tailed Student's *t*-test. The horizontal line indicates the Bonferroni-corrected genome-wide significance threshold ($\alpha = 0.05$) in a and b.

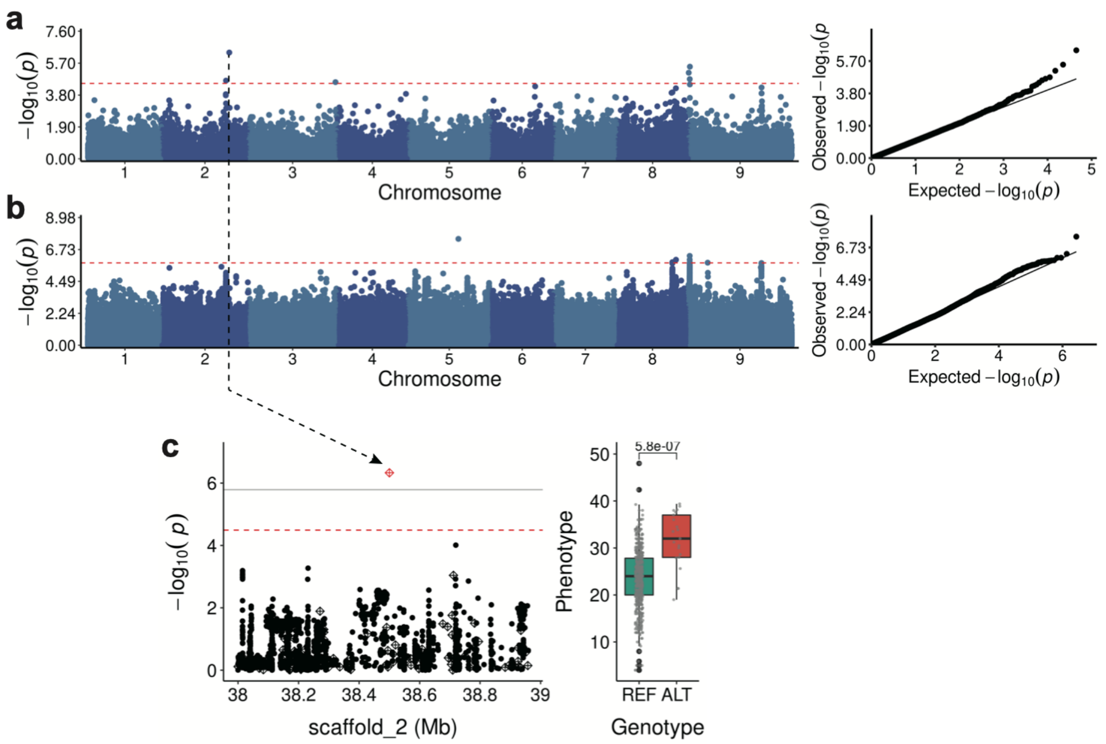

Supplementary Fig. 16 Genome-wide association study of peduncle length (PL). a, Manhattan plot and QQ-plot of GWAS result of PL using SV markers. Red dashed line is genome-wide significant threshold (p=3.22×10^{-5}). **b,** Manhattan plot and QQ-plot of GWAS result of PL using SNP markers. Red dashed line indicates genome-wide significant threshold (p=1.63×10^{-6}). **c,** Scatter plot of the peak-SV in chromosome 4 and boxplot of PL in different accessions carrying different alleles. Red diamonds and black points indicate PAVs significant associated with PL and SNPs association-level (-$\log_{10}(p)$) with PL, respectively. The horizontal line indicates the Bonferroni-corrected genome-wide significance threshold ($\alpha = 1$, and $\alpha = 0.05$). Numbers of samples for REF and ALT in boxplots of **c** are 132 and 529, respectively. In boxplots, the 25% and 75% quartiles are shown as lower and upper edges of boxes, respectively, and central lines denote the median. The whiskers extend to 1.5 times the inter-quartile range. Data beyond the end of the whiskers are displayed as black dots. *P*-values were computed from two-tailed Student's *t*-test. The horizontal line indicates the Bonferroni-corrected genome-wide significance threshold ($\alpha = 0.05$) in a and b.

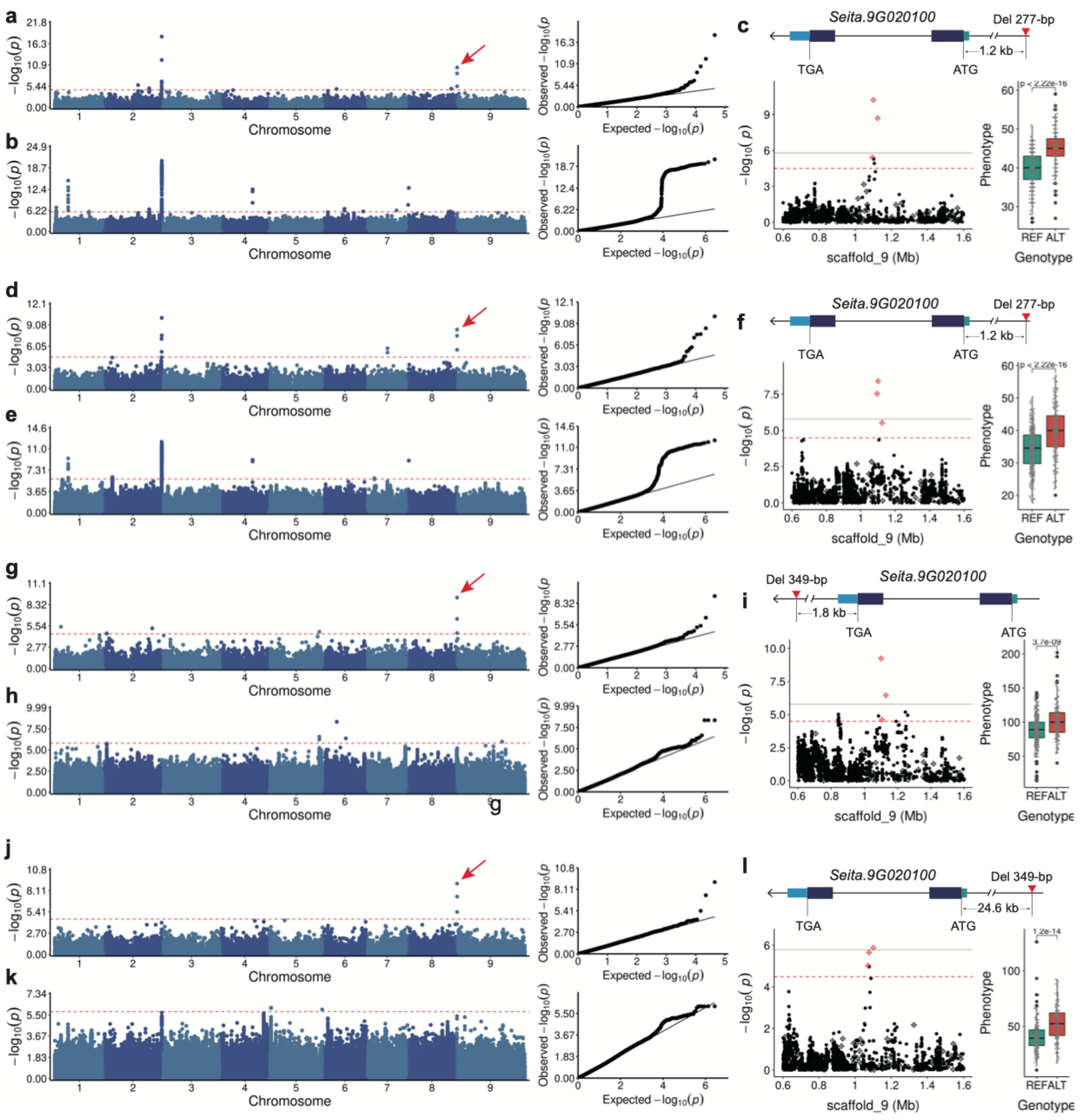

Supplementary Fig. 17 ***Seita.9G020100*** **associated with multiple key traits in foxtail millet, which only could be detected by SV-GWAS. a-b,** Manhattan plot and QQ-plot of GWAS results of heading date using SV (**a**) and SNP (**b**) markers. **d-e,** Manhattan plot and QQ-plot of GWAS results of leaf length using SV (**d**) and SNP (**e**) markers. **g-h,** Manhattan plot and QQ-plot of GWAS results of primary branch number using SV (**g**) and SNP (**h**) markers. **j-k,** Manhattan plot and QQ-plot of GWAS results of straw weight using SV (**j**) and SNP (**k**) markers. The SV and gene structure, scatter plot around peak-SV, and the boxplot of the corresponding phenotypes in different alleles of peak-SV for heading date (**c**), leaf length(**f**), primary branch number (**i**) and straw weight (**l**) are also shown. Numbers of samples for REF in boxplots of **c,f,i,l** are 436, 437, 474 and 223, respectively. Numbers of samples for ALT in boxplots of **c,f,i,l** are 207,215,184 and 140, respectively. In boxplots, the 25% and 75% quartiles are shown

as lower and upper edges of boxes, respectively, and central lines denote the median. The whiskers extend to 1.5 times the inter-quartile range. Data beyond the end of the whiskers are displayed as black dots. *P*-values were computed from two-tailed Student's *t*-test. The horizontal line indicates the Bonferroni-corrected genome-wide significance threshold ($\alpha = 0.05$) in a, b, d, e, g, h, j, and k. The horizontal line in c, f, i and l indicates the Bonferroni-corrected genome-wide significance threshold ($\alpha = 1$ and $\alpha = 0.05$).

Supplementary References

1. Pickrell, J. & Pritchard, J. Inference of population splits and mixtures from genome-wide allele frequency data. *Nat Prec* 1–1 (2012) doi:10.1038/npre.2012.6956.1.
2. Maier, R., Flegontov, P., Flegontova, O., Changmai, P. & Reich, D. On the limits of fitting complex models of population history to genetic data. 2022.05.08.491072 Preprint at https://doi.org/10.1101/2022.05.08.491072 (2022).
3. Refoyo-Martínez, A. *et al.* Identifying loci under positive selection in complex population histories. *Genome Res.* **29**, 1506–1520 (2019).
4. Tanabe, S. *et al.* A Novel Cytochrome P450 Is Implicated in Brassinosteroid Biosynthesis via the Characterization of a Rice Dwarf Mutant, dwarf11, with Reduced Seed Length. *The Plant Cell* **17**, 776–790 (2005).
5. Umemura, K. *et al.* Contribution of salicylic acid glucosyltransferase, OsSGT1, to chemically induced disease resistance in rice plants. *The Plant Journal* **57**, 463–472 (2009).
6. Lo, S.-F. *et al.* A Novel Class of Gibberellin 2-Oxidases Control Semidwarfism, Tillering, and Root Development in Rice. *The Plant Cell* **20**, 2603–2618 (2008).
7. Dai, X., Wang, Y., Yang, A. & Zhang, W.-H. OsMYB2P-1, an R2R3 MYB Transcription Factor, Is Involved in the Regulation of Phosphate-Starvation Responses and Root Architecture in Rice. *Plant Physiology* **159**, 169–183 (2012).
8. She, K.-C. *et al.* A Novel Factor FLOURY ENDOSPERM2 Is Involved in Regulation of Rice Grain Size and Starch Quality. *The Plant Cell* **22**, 3280–3294 (2010).
9. Alonge, M. *et al.* Major impacts of widespread structural variation on gene expression and crop improvement in tomato. *Cell* **182**, 145-161. e23 (2020).
