# Supplementary material for: A graph-based genome and pan-genome variation of the model plant Setaria
Source: Nat Genet. 2023 Jun 8;55(7):1232–42. doi: 10.1038/s41588-023-01423-w (PMC10335933; doi:10.1038/s41588-023-01423-w)
Supplement: Supplementary file 2 — Reporting Summary [file 41588_2023_1423_MOESM2_ESM.pdf]

nature portfolio

Corresponding author(s): Xianmin Diao, Michael Purugganan, and Guanqing Jia

Last updated by author(s): Mar 20, 2023

Reporting Summary

Nature Portfolio wishes to improve the reproducibility of the work that we publish. This form provides structure for consistency and transparency in reporting. For further information on Nature Portfolio policies, see our Editorial Policies and the Editorial Policy Checklist.

Statistics

For all statistical analyses, confirm that the following items are present in the figure legend, table legend, main text, or Methods section.

n/a	Confirmed	
☐	☒	The exact sample size (*n*) for each experimental group/condition, given as a discrete number and unit of measurement
☐	☒	A statement on whether measurements were taken from distinct samples or whether the same sample was measured repeatedly
☐	☒	The statistical test(s) used AND whether they are one- or two-sided<br>*Only common tests should be described solely by name; describe more complex techniques in the Methods section.*
☒	☐	A description of all covariates tested
☐	☒	A description of any assumptions or corrections, such as tests of normality and adjustment for multiple comparisons
☐	☒	A full description of the statistical parameters including central tendency (e.g. means) or other basic estimates (e.g. regression coefficient) AND variation (e.g. standard deviation) or associated estimates of uncertainty (e.g. confidence intervals)
☐	☒	For null hypothesis testing, the test statistic (e.g. *F*, *t*, *r*) with confidence intervals, effect sizes, degrees of freedom and *P* value noted<br>*Give P values as exact values whenever suitable.*
☒	☐	For Bayesian analysis, information on the choice of priors and Markov chain Monte Carlo settings
☒	☐	For hierarchical and complex designs, identification of the appropriate level for tests and full reporting of outcomes
☒	☐	Estimates of effect sizes (e.g. Cohen's *d*, Pearson's *r*), indicating how they were calculated

Our web collection on statistics for biologists contains articles on many of the points above.

Software and code

Policy information about availability of computer code

Data collection	Data were sequenced from PacBio Sequel and Illumina NovaSeq 6000. Publicly available data were downloaded and used.
Data analysis	We used publicly available and appropriately cited software as described. No commercial software or code was used in this study. Software are listed as follows: fastp (v0.23.0), BWA (v0.7.12-r1039),SAMtools (v1.7),GATK (v4.1.4),SnpEff (v5.0),vg toolkit (v1.28.0),MEGA-CC (v10.1.8),SNPhylo (v2018-09-01),IQ-TREE (v2.1.2),ADMIXTURE (v1.3.0),PLINK (v.1.90),Admixtools (v2.0),TreeMix (v1.13),CANU (v2.2),HERA (v1.0),Pilon (version 1.22), IrysSolve (v3.5_12162019, https://bionanogenomics.com/support/software-downloads/),Mummer (v4.0),BUSCO (v5.2.0),LTR_retriever (v2.9.0),Merqury (v1.3),LTR_FINDER (v1.05),RepeatModeler (v4.0.6),RepeatMasker (v1.0.10),EDTA (v1.9.4),GMAP (v2015-09-21),diamond (v0.9.25),OrthoFinder (v2.3.12),SyRI(v1.2),minimap2 (v2.21-r1071),EMMAX (v20120210),R (v4.03),VCFtools (v 0.1.17),XP-CLR(v1.1.2,https://github.com/hardingnj/xpclr),CropGBM(v1.1.2).<br>Custom codes are available at github: https://github.com/qiangh06/Setaria-pan-genome and Zenodo: https://doi.org/10.5281/zenodo.7743007.

For manuscripts utilizing custom algorithms or software that are central to the research but not yet described in published literature, software must be made available to editors and reviewers. We strongly encourage code deposition in a community repository (e.g. GitHub). See the Nature Portfolio guidelines for submitting code & software for further information.

Data

Policy information about availability of data

All manuscripts must include a data availability statement. This statement should provide the following information, where applicable:

- Accession codes, unique identifiers, or web links for publicly available datasets
- A description of any restrictions on data availability
- For clinical datasets or third party data, please ensure that the statement adheres to our policy

All long-read sequencing data and three Bionano cmap files have been deposited in National Center for Biotechnology Information database under accession code BioProject: PRJNA675302. All 110 assembled genomes and annotations were deposited at https://www.zenodo.org/record/7367881. 1004 NGS re-sequencing data generated have been deposited in the NCBI database under accession code BioProject: PRJNA841774 and PRJNA842100. Other 294 foxtail millet and 594 green foxtail whole genome sequencing data were downloaded from NCBI (BioProject PRJNA636263, PRJNA560514 and PRJNA265547).The phenotypes used in GWAS and GS studies have been deposited in https://doi.org/10.5281/zenodo.7755340.

Human research participants

Policy information about studies involving human research participants and Sex and Gender in Research.

Reporting on sex and gender	*Use the terms sex (biological attribute) and gender (shaped by social and cultural circumstances) carefully in order to avoid confusing both terms. Indicate if findings apply to only one sex or gender; describe whether sex and gender were considered in study design whether sex and/or gender was determined based on self-reporting or assigned and methods used. Provide in the source data disaggregated sex and gender data where this information has been collected, and consent has been obtained for sharing of individual-level data; provide overall numbers in this Reporting Summary. Please state if this information has not been collected. Report sex- and gender-based analyses where performed, justify reasons for lack of sex- and gender-based analysis.*
Population characteristics	*Describe the covariate-relevant population characteristics of the human research participants (e.g. age, genotypic information, past and current diagnosis and treatment categories). If you filled out the behavioural & social sciences study design questions and have nothing to add here, write "See above."*
Recruitment	*Describe how participants were recruited. Outline any potential self-selection bias or other biases that may be present and how these are likely to impact results.*
Ethics oversight	*Identify the organization(s) that approved the study protocol.*

Note that full information on the approval of the study protocol must also be provided in the manuscript.

Field-specific reporting

Please select the one below that is the best fit for your research. If you are not sure, read the appropriate sections before making your selection.

☒ Life sciences ☐ Behavioural & social sciences ☐ Ecological, evolutionary & environmental sciences

For a reference copy of the document with all sections, see nature.com/documents/nr-reporting-summary-flat.pdf

Life sciences study design

All studies must disclose on these points even when the disclosure is negative.

Sample size	We selected 110 representative Setaria accessions, including 35 wild, 40 landrace, and 35 modern cultivated accessions. The logic of this selection was based on phylogenetic relationships and geographic distribution, breeding and/or research contribution, and subgroups distributions to ensure that they are representative of genetic diversity within foxtail millet and green foxtail.
Data exclusions	No samples were excluded in this study. Filters applied to eliminate low-quality sequencing data and genetic variants were properly described in the Method section.
Replication	Five biological replicates were used in the qRT-PCR experiment. Three independent T3 transgenic lines were generated for the estimation of thousand grain weight, grain width, and grain length, in which three independent wild-type plants were also measured. All replications were successful and were used.
Randomization	For each foxtail millet or green foxtail individual, the sampling process for genome DNA/RNA sequencing was randomly conducted. All WT and transgenic plants were exposed to the same growth condition and treatment. For phenotype data collection at GWAS and GS studies, we randomly selected 3-5 plants for data collection per accessions in the filed.
Blinding	Blinding is not necessary for genome sequencing and assembly, since the investigators know which Setaria accessions they were handling. The investigators were blinded to group allocation during collecting data from WT and transgenic lines.

Reporting for specific materials, systems and methods

We require information from authors about some types of materials, experimental systems and methods used in many studies. Here, indicate whether each material, system or method listed is relevant to your study. If you are not sure if a list item applies to your research, read the appropriate section before selecting a response.

Materials & experimental systems

n/a	Involved in the study
☒	☐ Antibodies
☒	☐ Eukaryotic cell lines
☒	☐ Palaeontology and archaeology
☒	☐ Animals and other organisms
☒	☐ Clinical data
☒	☐ Dual use research of concern

Methods

n/a	Involved in the study
☒	☐ ChIP-seq
☒	☐ Flow cytometry
☒	☐ MRI-based neuroimaging
